# A Prediction Model of the Capillary Pressure *J*-Function

**DOI:** 10.1371/journal.pone.0162123

**Published:** 2016-09-07

**Authors:** W. S. Xu, P. Y. Luo, L. Sun, N. Lin

**Affiliations:** 1 State Key of Oil and Gas Reservoir Geology and Exploitation, Southwest Petroleum University, Chengdu, Sichuan, 610500, People’s Republic of China; 2 Tarim Oilfield Company, PetroChina, Korla, Xinjiang, 841000, People’s Republic of China; University of Arizona, UNITED STATES

## Abstract

The capillary pressure *J*-function is a dimensionless measure of the capillary pressure of a fluid in a porous medium. The function was derived based on a capillary bundle model. However, the dependence of the *J*-function on the saturation *S*_w_ is not well understood. A prediction model for it is presented based on capillary pressure model, and the *J*-function prediction model is a power function instead of an exponential or polynomial function. Relative permeability is calculated with the *J*-function prediction model, resulting in an easier calculation and results that are more representative.

## 1. Introduction

A capillary pressure curve is determined based on the measurements of a small rock sample, and therefore, it represents only one part of a reservoir. As a method to compare capillary pressure data for a range of practical applications, Leverett [1] proposed the semi-empirical *J*-function

$$J(S_{w})=\frac{p_{c}(S_{w})}{\sigma \cos\theta }\sqrt{\frac{k}{\phi }}$$ (1)

where *J(S*_*w*_*)* is the *J*-function, *S*_*w*_ is the wetting-phase saturation, *p*_*c*_(*S*w) is the capillary pressure, *σ* is the interfacial tension (IFT), *θ* is the contact angle, *k* is permeability, and *φ* is porosity.

The *J*-function, which synthesizes the fluid IFT, wettability, permeability, and porosity, is used to represent the characteristics of the reservoir capillary pressure curve. It is an effective method for analyzing data on capillary pressure. Leverett postulated that the *J*-function of similar lithology has universal significance, and in most cases, all capillary pressures of a reservoir processed with the *J*-function are simplified to a single monotonic curve. Brown [2] studied the *J*-function in more detail and concluded that the *J*-function of the same strata and lithology provides a better comparison, which supports the view of Rose and Bruce [3]. Therefore, the *J*-function can be used to represent the capillary pressure curve of a reservoir.

However, the initial *J*-function expression was simply proposed but not derived specifically by Leverett [1]. Though some researchers [4–7] attempted to modify the *J*-function, such as with the Gao fractal model [7], their models have no fundamental improvement because the origin of the initial *J*-function is unknown.

Considering the research background and technical level of the field at the time that the *J*-function was proposed, we conclude that the *J*-function formula was derived based on the capillary bundle model. Because only capillary pressure varies in the *J*-function expression, the *J*-function prediction formula starts with a mathematical model. The fitting trend for *J*-function is a power function rather than an exponential or polynomial one through comprehensive investigations of the capillary pressure quantitative relation. Finally, relative permeability is calculated with the *J*-function prediction formula. The calculation is easier than with earlier formulas, and its results are more representative.

## 2. Realization of *J*-Function Derivation

Research shows that rocks should be described using the direct-mapping pore network model [8–12] However, owing to the research background and technical level of the Leverett era, rocks were described using the bundle-of-tubes model or the capillary bundle model. The capillary bundle model was widely applied as the classical model [13–15] such as in the derived Kozeny–Carman model [16]. Thus, the capillary bundle model was used to derive the *J*-function.

**Fig 1:**
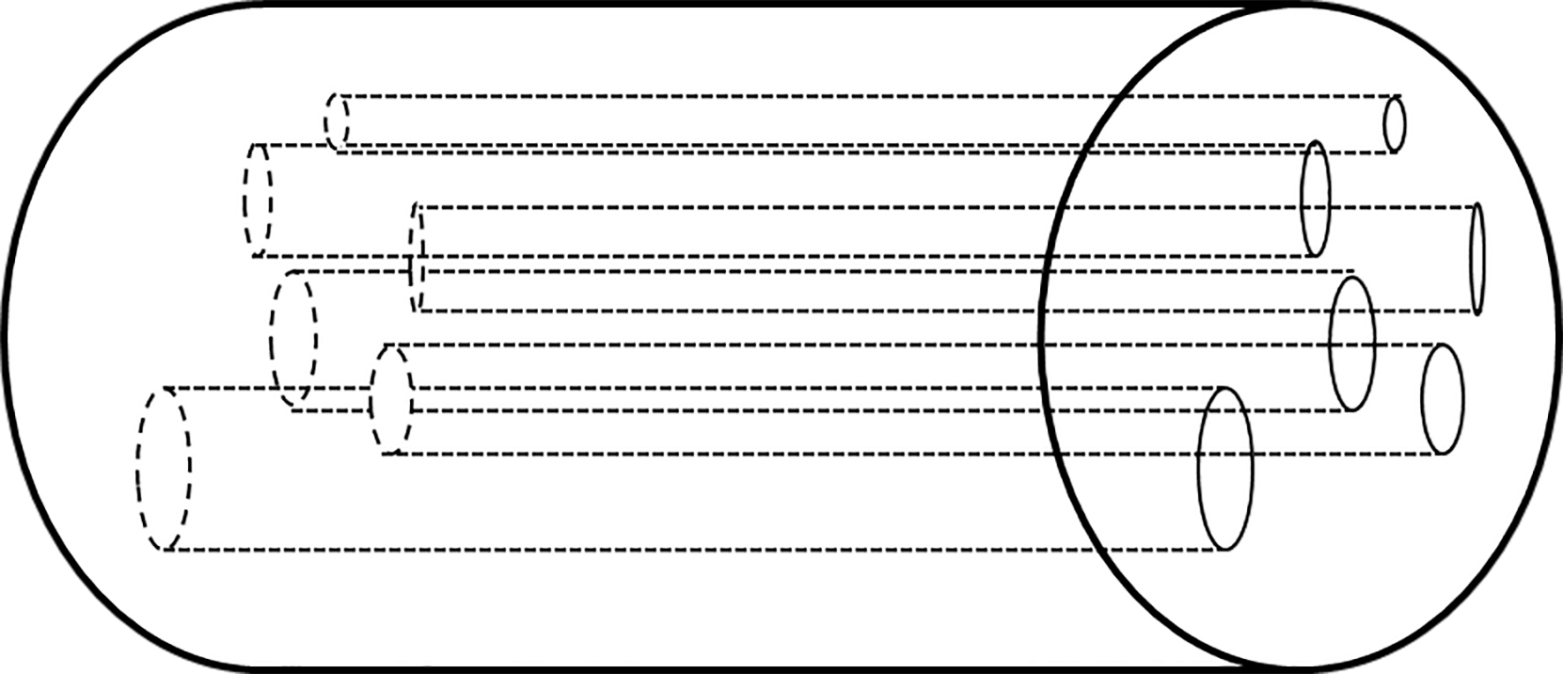
Capillary bundle model.

When the capillary bundle model (Fig 1) is used to describe a rock, the flow of one capillary tube according to the Poiseuille equation (*n* capillary tubes) is given as

$$q=\frac{n\pi r^{4}}{8\mu l_{1}}\Delta p$$ (2)

where *q* is the flow, *r* is the capillary tube radius, *μ* is the viscosity, *l*_*1*_ is the length of the capillary tube, and Δ*p* is the pressure difference.

The porosity of the capillary bundle model core is

$$\phi =\frac{n\pi r^{2}l_{1}}{Al_{1}}=\frac{n\pi r^{2}}{A}$$ (3)

And according to Darcy’s law, permeability is given as

$$k=\frac{q\mu l_{2}}{A\Delta p}$$ (4)

where *k* is permeability, *A* is core area and *l*_*2*_ is the length of the core.

Substituting Eq 2 into Eq 4, we get

$$k=\frac{l_{2}}{8l_{1}}\cdot \frac{n\pi r^{4}}{A}$$ (5)

Substituting Eq 3 into Eq 5, and re-arranging the expression, we get

$$r^{2}=\frac{8k}{\phi }\frac{l_{1}}{l_{2}}$$ (6)

where *l*_*1*_ is not equal to *l*_*2*_, and the ratio between them is referred to as tortuosity. Tortuosity is constant for a certain core; hence,

$$r=C\sqrt{\frac{k}{\phi }}$$ (7)

Combining Eq 7 with the definition of capillary pressure,

$$p_{c}=\frac{2\sigma \cos\theta }{r}$$ (8)

the dimensionless capillary pressure *J*-function (Eq 1**)** can be derived.

## 3. Prediction Model about the Dependence of *J*-Function on *S*_*w*_

In recent years, rocks have been described using an interconnected network model, which is a significant improvement on the capillary bundle model because it can accurately predict the capillary pressure curve and simulate drainage and the imbibition process [9–10,12]. However, the network model has multiple and complex pore structure parameters; therefore, it is difficult to find analytical expressions for the *J* curve based on network models. Considering that only capillary pressure is variable in the *J*-function expression, the prediction about the dependence of *J*-function on *S*_*w*_ should start with the dependence of capillary pressure on *S*_*w*_, that’s called the capillary pressure mathematical model.

The main mathematical models for capillary pressure are the Thomeer [17], Brooks–Corey [18], van [19], and the three-constant hyperbolic models [20–21]. The Brooks–Corey model is widely used owing to its accuracy [22–25], and it is expressed as

$$S_{\text{e}}={\left(\frac{P_{d}}{P_{c}}\right)}^{\lambda }$$ (9)

Or

$$p_{c}=p_{d}{\left(S_{e}\right)}^{-\frac{1}{\lambda }}$$ (10)

where *p*_*d*_ is the threshold pressure, *S*_e_ is the effective saturation or the normalized saturation, and *λ* is the pore size distribution index; *S*_*e*_ is defined as

$$S_{e}=\frac{S_{\text{w}}-S_{\text{r}}}{1-S_{\text{r}}}$$ (11)

where *S*_*w*_ is the wetting phase saturation and *S*_*r*_ is the minimum wetting phase saturation.

Substituting Eq 10 into Eq 1, we get

$$J(S_{e})=\frac{p_{d}\sqrt{\frac{k}{\phi }}}{\sigma \cos\theta }S_{e}^{-\frac{1}{\lambda }}$$ (12)

which is further simplified as

$$J(S_{e})=AS_{e}^{B}$$ (13)

where $A=\frac{p_{d}\sqrt{\frac{k}{\phi }}}{\sigma \cos\theta }$, $B=-\frac{1}{\lambda }$.

Therefore, the fitting trend for the average capillary pressure *J*-function is a power function rather than an exponential or a polynomial one.

**Fig 2:**
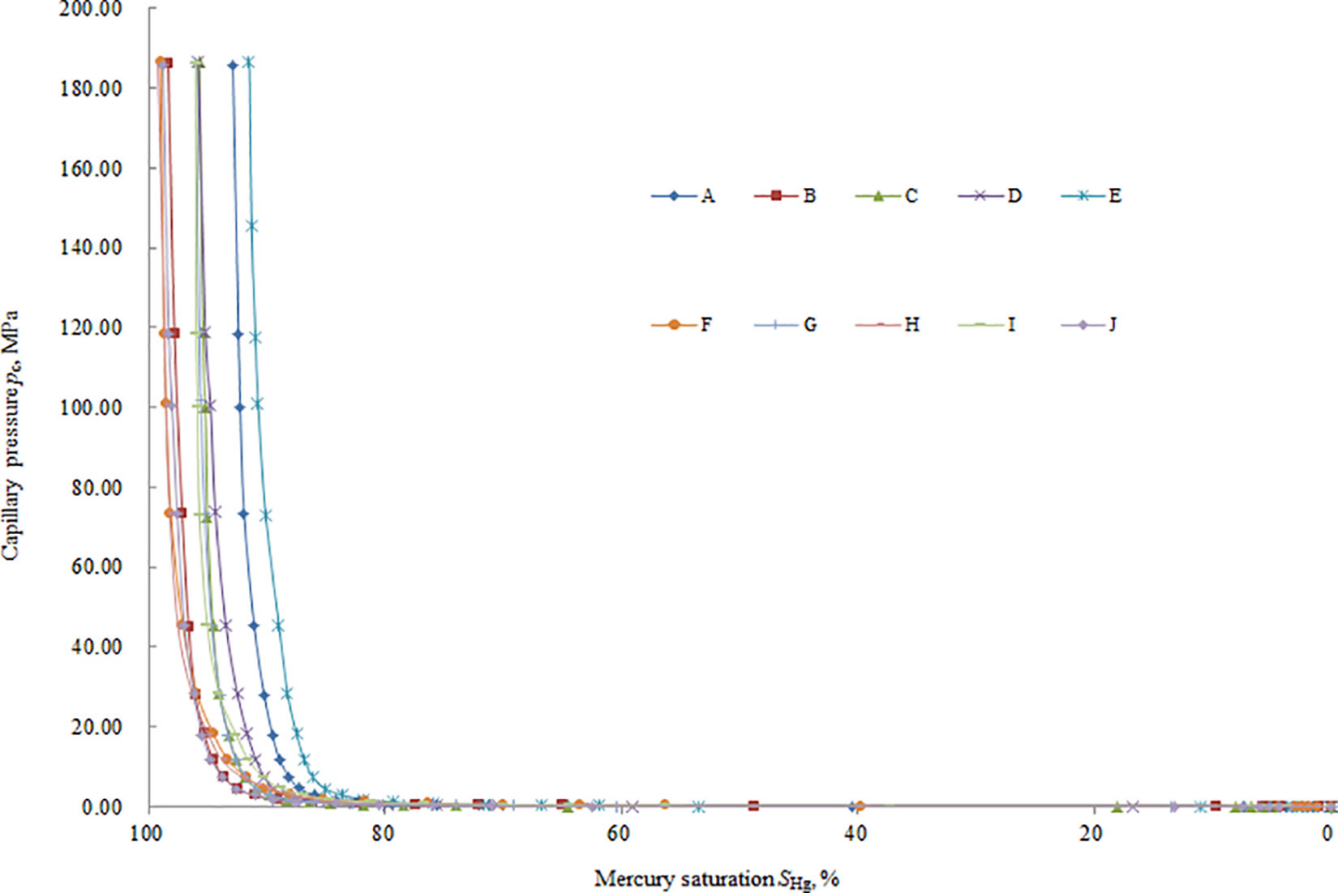
Capillary pressure raw data.

**Fig 3:**
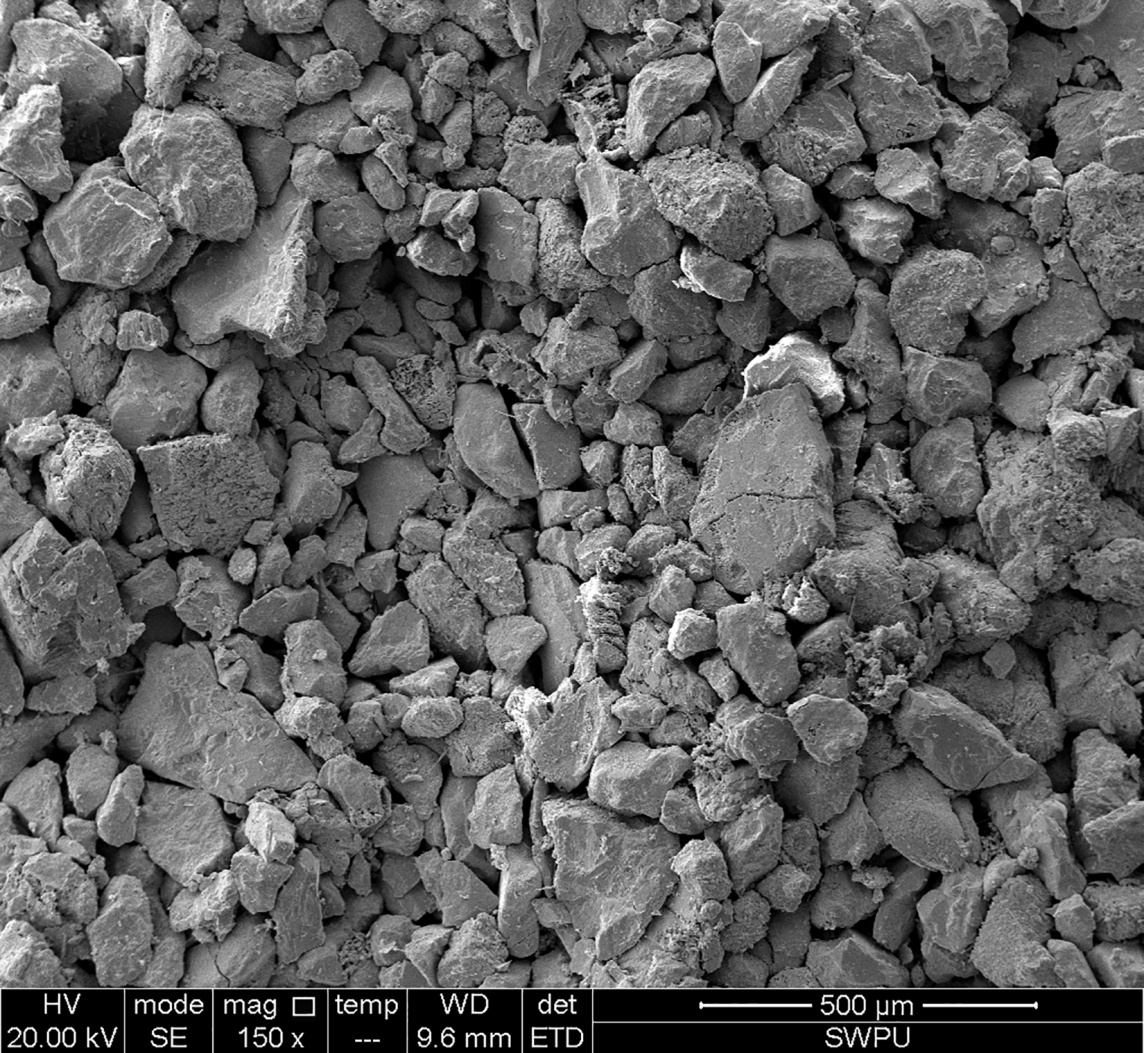
SEM image of rock lithology (Sample F in S1 Data).

**Fig 4:**
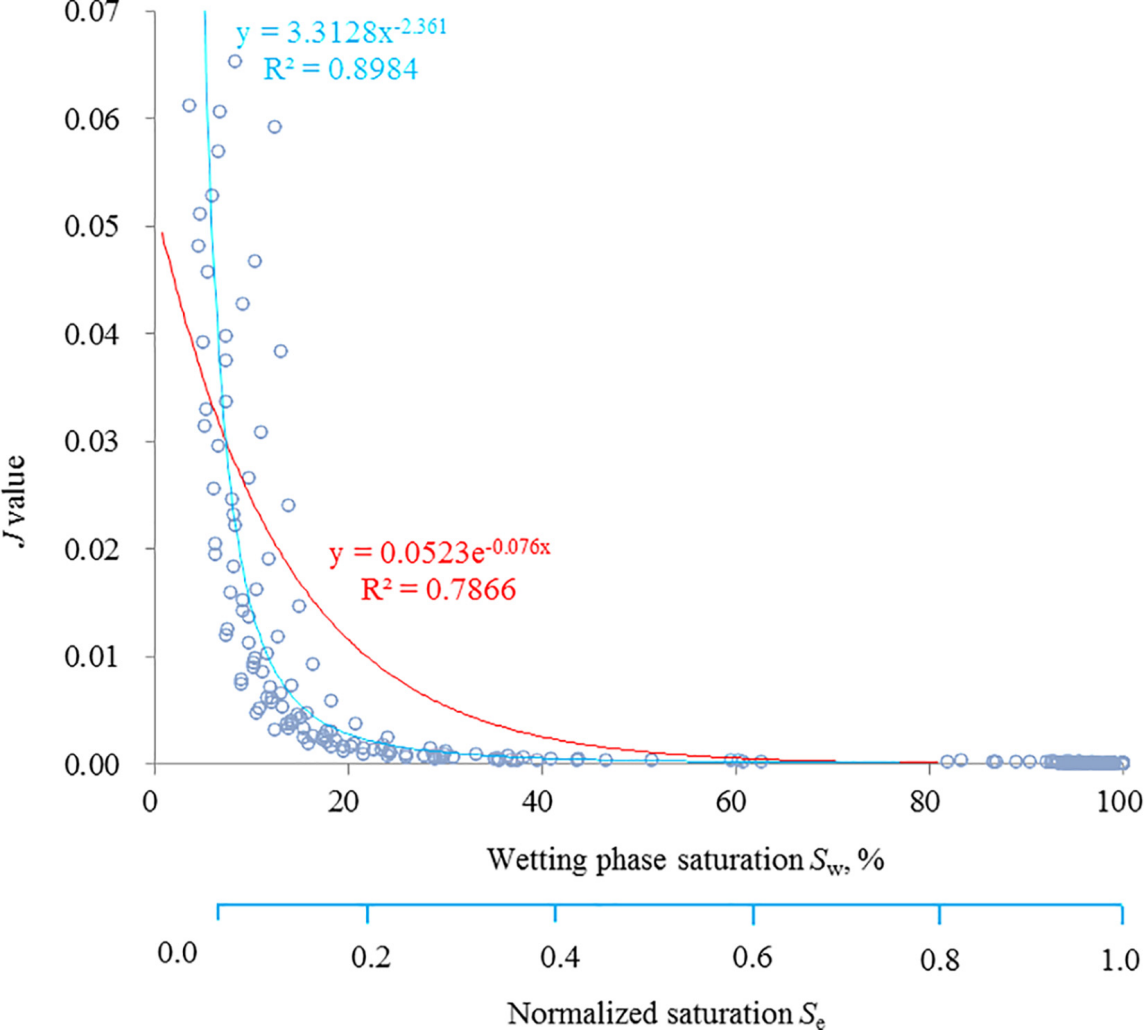
Fitting chart.

Because the *J*-function of the same strata and lithology provides a better comparison [2–3,16,24], 10 representative rocks from the WJ Well, shan 2–2 strata of the ZY oil field in China were selected for the practical calculation. The capillary pressure raw data (S1 Data) are shown in Fig 2, and they have a sandstone lithology (Fig 3). The practical calculation about the dependence of *J*-function on the wetting phase saturation *S*_*w*_ for the ten rocks is shown in Fig 4.

Fig 4 shows that the coefficient of determination (*R*^*2*^) of the power form is greater than that of the exponential form; hence, the fitting precision of the power form is more accurate. This verifies the correctness of the *J*-function prediction model.

## 4. Application of the *J*-Function Prediction Model

The main application of the *J*-function is determining the average capillary pressure, which is the representative capillary pressure of a reservoir layer. The relative permeability calculated with the average capillary pressure obtained using the *J*-function average is more representative. The calculation can be further simplified and made more convenient by substituting the *J*-function expression into the empirical formula of relative permeability. The theoretical expression of the relative permeability calculated with the *J*-function prediction model is given below.

Because the Purcell [26] relative permeability model was derived based on the capillary bundle model, which is far from the true rock pore structure, Burdine [27] introduced tortuosity into the Purcell model to improve precision. The Burdine model is widely applied as a classical model [16,27–29]. The wetting phase relative permeability model of Burdine [16,27,29] is

$$k_{rw}(S_{e})={\left(S_{e}\right)}^{2}\frac{\int_{0}^{S_{e}}\frac{1}{p_{c}^{2}(S_{e})}dS_{e}}{\int_{0}^{1}\frac{1}{p_{c}^{2}(S_{e})}dS_{e}}$$ (14)

The *J*-function prediction formula is

$$J(S_{e})=\frac{p_{c}(S_{e})}{\sigma \cos\theta }\sqrt{\frac{k_{av}}{\phi_{av}}}=A{\left(S_{e}\right)}^{B}$$ (15)

The average capillary pressure is

$$p_{c}(S_{e})=\sigma \cos\theta \sqrt{\frac{\phi_{av}}{k_{av}}}A{\left(S_{e}\right)}^{B}$$ (16)

which is further simplified as

$$p_{c}(S_{e})=DA{\left(S_{e}\right)}^{B}$$ (17)

and *D* is

$$D=\sigma \cos\theta \sqrt{\frac{\phi_{av}}{k_{av}}}$$ (18)

where *k*_*av*_ is the average permeability and *φ*_*av*_ is the average porosity.

Substituting Eq 16 into Eq 14, the wetting phase relative permeability of the Burdine model becomes

$$k_{rw}(S_{e})={\left(S_{e}\right)}^{2}\frac{\int_{0}^{S_{e}}\frac{1}{{\left(DA\right)}^{2}{\left(S_{e}\right)}^{2B}}dS_{e}}{\int_{0}^{1}\frac{1}{{\left(DA\right)}^{2}{\left(S_{e}\right)}^{2B}}dS_{e}}$$ (19)

The calculation result of Eq 19 is

$$k_{rw}(S_{e})={\left(S_{e}\right)}^{3-2B}$$ (20)

Similarly, the calculation result of the non-wetting phase relative permeability model is

$$\{\begin{array}{l} k_{rnw}(S_{e})={\left(1-S_{e}\right)}^{2}\frac{\int_{S_{e}}^{1}\frac{1}{p_{c}^{2}(S_{e})}dS_{e}}{\int_{0}^{1}\frac{1}{p_{c}^{2}(S_{e})}dS_{e}}\\ k_{rnw}(S_{e})={\left(1-S_{e}\right)}^{2}[1-{\left(S_{e}\right)}^{1-2B}] \end{array}$$ (21)

Therefore, a set of new and more representative saturation functions, which are the basic input parameters for the Eclipse SCAL Section, can be composed of the analytic expressions discussed in this section. This new set of saturation functions is summarized as follows:

$$\{\begin{array}{l} J(S_{e})=A{\left(S_{e}\right)}^{B}\\ k_{rw}(S_{e})={\left(S_{e}\right)}^{3-2B}\\ k_{rnw}(S_{e})={\left(1-S_{e}\right)}^{2}[1-{\left(S_{e}\right)}^{1-2B}] \end{array}$$ (22)

## 5. Conclusions

1. The Leverett *J*-function is derived based on the capillary bundle model.
2. The prediction *J*-function model is a power function that points the way for its application.
3. The empirical formula of the relative permeability calculation is easier to use and more convenient with the *J*-function prediction formula than with the earlier formula; the calculated results are more representative.

## Supporting Information

S1 Data Capillary pressure raw data. (XLSX)
